# The novel fosfomycin resistance gene fosY is present on a genomic island in CC1 methicillin-resistant *Staphylococcus aureus*

**DOI:** 10.1080/22221751.2022.2058421

**Published:** 2022-04-20

**Authors:** Yiyi Chen, Shujuan Ji, Lu Sun, Haiping Wang, Feiteng Zhu, Mengzhen Chen, Hemu Zhuang, Zhengan Wang, Shengnan Jiang, Yunsong Yu, Yan Chen

**Affiliations:** a Department of Infectious Diseases, Sir Run Run Shaw Hospital, Zhejiang University School of Medicine, Hangzhou, People’s Republic of China; b Key Laboratory of Microbial Technology and Bioinformatics of Zhejiang Province, Hangzhou, People’s Republic of China; c Regional Medical Center for National Institute of Respiratory Diseases, Sir Run Run Shaw Hospital, Zhejiang University School of Medicine, Hangzhou, People’s Republic of China

**Keywords:** Fosfomycin, MRSA, *fosY*, resistance island, resistant gene

## Abstract

Fosfomycin has gained attention as a combination therapy for methicillin-resistant *Staphylococcus aureus* infections. Hence, the detection of novel fosfomycin-resistance mechanisms in *S. aureus* is important. Here, the minimal inhibitory concentrations (MICs) of fosfomycin in CC1 methicillin-resistant *S. aureus* were determined. The pangenome analysis and comparative genomics were used to analyse CC1 MRSA. The gene function was confirmed by cloning the gene into pTXΔ. A phylogenetic tree was constructed to determine the clustering of the CC1 strains of *S. aureus.* We identified a novel gene, designated *fosY*, that confers fosfomycin resistance in *S. aureus*. The FosY protein is a putative bacillithiol transferase enzyme sharing 65.9–77.5% amino acid identity with FosB and FosD, respectively. The function of *fosY* in decreasing fosfomycin susceptibility was confirmed by cloning it into pTXΔ. The pTX-*fosY* transformant exhibited a 16-fold increase in fosfomycin MIC. The bioinformatic analysis showed that *fosY* is in a novel genomic island designated RI*_fosY_* (for “resistance island carrying *fosY*”) that originated from other species. The global phylogenetic tree of ST1 MRSA displayed this *fosY*-positive ST1 clone, originating from different regions, in the same clade. The novel resistance gene in the fos family, *fosY*, and a genomic island, RI*_fosY_*, can promote cross-species gene transfer and confer resistance to CC1 MRSA causing the failure of clinical treatment. This emphasises the importance of genetic surveillance of resistance genes among MRSA isolates.

## Introduction

Fosfomycin exhibits broad-spectrum activity against both gram-positive and gram-negative bacteria by interfering with the first committed step of peptidoglycan synthesis [1]. The lack of new antibiotics and the development of resistance in bacteria have revived the interest of clinicians in older drugs such as fosfomycin. In recent years, fosfomycin in combination with other antibiotics has been used to treat infections by methicillin-resistant *Staphylococcus aureus* (MRSA), an important pathogen [2].

Mutations in the genes encoding fosfomycin transporters such as *glpT* and *uphT* or the target enzyme MurA and the acquisition of the fosfomycin-modifying enzymes are two major mechanisms of fosfomycin resistance [3]. The main enzymes that inactivate fosfomycin include three types of metalloenzymes with different substrates and two kinases [4]. In low-GC gram-positive bacteria, fosfomycin-modifying enzymes catalyse the reaction between bacillithiol and fosfomycin [5]. To date, two genes *fosB* and *fosD,* encoding bacillithiol-S-transferases, have been identified in staphylococci [6,7].

*FosB*, first discovered in the plasmids of *S. epidermidis* in 1990 [6], was later found in *S. aureus*. *FosD*, located in the plasmid of *S. aureus* [7], shows 78.9% nucleotide and 74.1% amino acid sequence identity with the fosfomycin-resistance determinant *fosB* carried by MRSA strains.

Multilocus sequence type 1 (ST1) MRSA isolates, also known as pulsed-field type USA400, were among the most prominent community-associated (CA) strains in the USA [8]. Recently, ST1 has been sporadically reported in other regions [9], including China [10]. As fosfomycin remains effective against community-onset MRSA [11], it has become an important alternative treatment for CA-MRSA infections. Therefore, the discovery of new resistance mechanisms in *S. aureus* is important.

In this study, we identified a novel gene, *fosY*, associated with fosfomycin resistance in genetic mobile elements in CC1 MRSA and characterised the gene function and surrounding structure of *fosY*. We also constructed a phylogenetic tree to clarify the characteristics of *fosY*-positive MRSA clone in China.

## Materials and methods

### Bacterial strains

*Staphylococcus aureus* isolates and clinical data were collected from 22 tertiary hospitals in 18 provinces and municipalities in China in a multicentre prospective study, and 471 MRSA isolates were previously subjected to next-generation sequencing, de novo assembly and MLST typing [12].

### Antimicrobial susceptibility testing

The minimal inhibitory concentrations (MICs) of fosfomycin were determined using the agar dilution method according to CLSI guidelines using Mueller–Hinton agar plates containing 25 mg/L glucose-6-phosphate. Susceptibility to other antibiotics was tested using the broth dilution method. Fosfomycin activity was interpreted based on the European Committee on Antimicrobial Susceptibility Testing [13] whereas the activities of other antibiotics were interpreted in accordance with the Clinical and Laboratory Standards Institute [14]. ATCC 29213 (*S. aureus*) was used as a quality control strain.

### Whole genome sequencing, comparative genomics analysis and phylogenetic analysis

The fosfomycin resistance gene detection was performed using web-based Center for Genomic Epidemiology (http://www.genomicepidemiology.org/). The mutations of chromosomal genes were confirmed via allele genes table in cgMLST generated in SeqSphere+ software version 4.1.9 (Ridom GmbH). The comparative genomics analysis of ST1 isolates in this study was performed using panaroo [15] to analyse the pangenome and detect new resistance determinant.

A multisequence alignment of the amino acid sequences of new protein and other fosfomycin-modifying enzymes which have been reported previously (Table S1) was conducted using ClustalX and the phylogenetic tree was constructed via the MEGA X [16] using the maximum likelihood method. The alignment of amino acid sequences was generated using ESPript 3.0 [17].

One of the ST1 strains N12HSA28 was selected randomly for further analysis. The Nanopore sequencing using a MinION sequencer (Oxford Nanopore Technologies, Oxford, UK) was performed. Hybrid assembly was performed using Unicycler with Illumina and Nanopore reads. The assembled contigs were annotated using the RAST server. Further sequence analysis was performed using Mauve and sequencing alignment software BLASTn (https://blast.ncbi.nlm.nih.gov/BlastAlign.cgi), a comparison map was generated using Easyfig2.1.

The maximum likelihood tree of ST1 *S. aureus* genomes included isolates in this study and those from public Genbank databases was constructed from a core genome alignment using Panaroo [15] with the tree generated by IQ-TREE 2 using the bootstrap method for test with 1000 replicates [18]. The phylogenetic tree was modified via iTOL (https://itol.embl.de/login.cgi). The information of public genomes was in Table S2.

### Cloning experiments

The *fosY* gene from N12HSA28 was amplified using PCR, digested with BamHI and MluI, and ligated into BamHI- and MluI-digested pTXΔ, a plasmid from *S. aureus* [19]. The recombinant plasmid pTX-*fosY* was transformed into *S. aureus* RN4220 via electroporation and successful transformants were selected with tetracycline (12.5 mg/L). The plasmid pTX16 was also introduced into the RN4220 strain as a control.

### Nucleotide sequence accession number

The *fosY* DNA sequence has been deposited in the Nucleotide database in NCBI under accession number MN961674.1. The genome of N12HSA28 was submitted to NCBI under accession number CP091523. The sequence of the resistance island carrying *fosY* (RI*_fosY_*) has been deposited in the genbank database under accession number OM925572.

## Results

### Identification of fosY in CC1 MRSA

A multicentre prospective study in China identified 11 MRSA strains belonging to STl. The fosfomycin MIC of these strains ranged from 0.5 to 16 mg/L, *fosB* and *fosD* were not present, and chromosomal mutations were not detected (Table 1). We compared the accessory genome of these strains and found an ORF of 423 bp, annotated as putative metallothiol transferase, in three strains whose fosfomycin MIC was ≥8 mg/L. The 423 bp ORF, encoding a protein of 140 amino acids, was designated as *fosY*. This novel gene exhibited 75.8% and 80.4% sequence identity with *fosB* in *S. epidermidis* [6] and *fosD* gene in *S. aureus* [7], respectively.

**Table 1.** Fosfomycin susceptibility testing of CC1-MRSA in this study.

Strains	Epidemiological investigation			Fosfomycin MIC (mg/L)	Molecular investigation			
	Province	Diseases	Sources		fosB/fosD	Mutations	fosY gene	MLST
N09CSA08	Guangdong	Rhinosinusitis	Secretion	0.5	–	–	–	1
N09CSA14	Guangdong	otitis	Ssecretion	0.5	–	–	–	1
N09CSA27	Guangdong	Pneumonia	Sputum	0.5	–	–	–	1
N09CSA29	Guangdong	UTI	Urine	0.5	–	–	–	1
N16CSA19	Henan	Otitis	Secretion	1	–	–	–	1
N16HSA11	Henan	SSTI	Pus	2	–	–	–	1
N23HSA01	Gansu	Respiratory infection	Sputum	4	–	–	–	1
N26HSA10	Guizhou	SSTI	Secretion	1	–	–	–	1
N12CSA41	Sichuan	Respiratory infection	Sputum	16	–	–	+	5537
N12HSA28	Sichuan	SSTI	Secretion	16	–	–	+	1
N24HSA04	Hainan	Respiratory infection	Sputum	16	–	–	+	1
N24HSA33	Hainan	Pneumonia	Sputum	8	–	–	+	1

Note: Mutations mean mutations in the chromosome genes as *glpT*, *uphT*, and *murA*.

Abbreviations: MIC: Minimum Inhibitory concentration; SSTI: Skin and Soft Tissue Infection; UTI: urinary tract infection.

Further screening of all sequenced isolates revealed another ST5537 strain, which was a single-locus variant of ST1 and classified as complex clone 1 (CC1), that also carried this gene. Table 1 presents basic information about the 12 strains. The isolates were scattered across six provinces. Most strains were collected from the secretion of SSTI patients or sputum of patients with respiratory infections.

**Figure 1.**
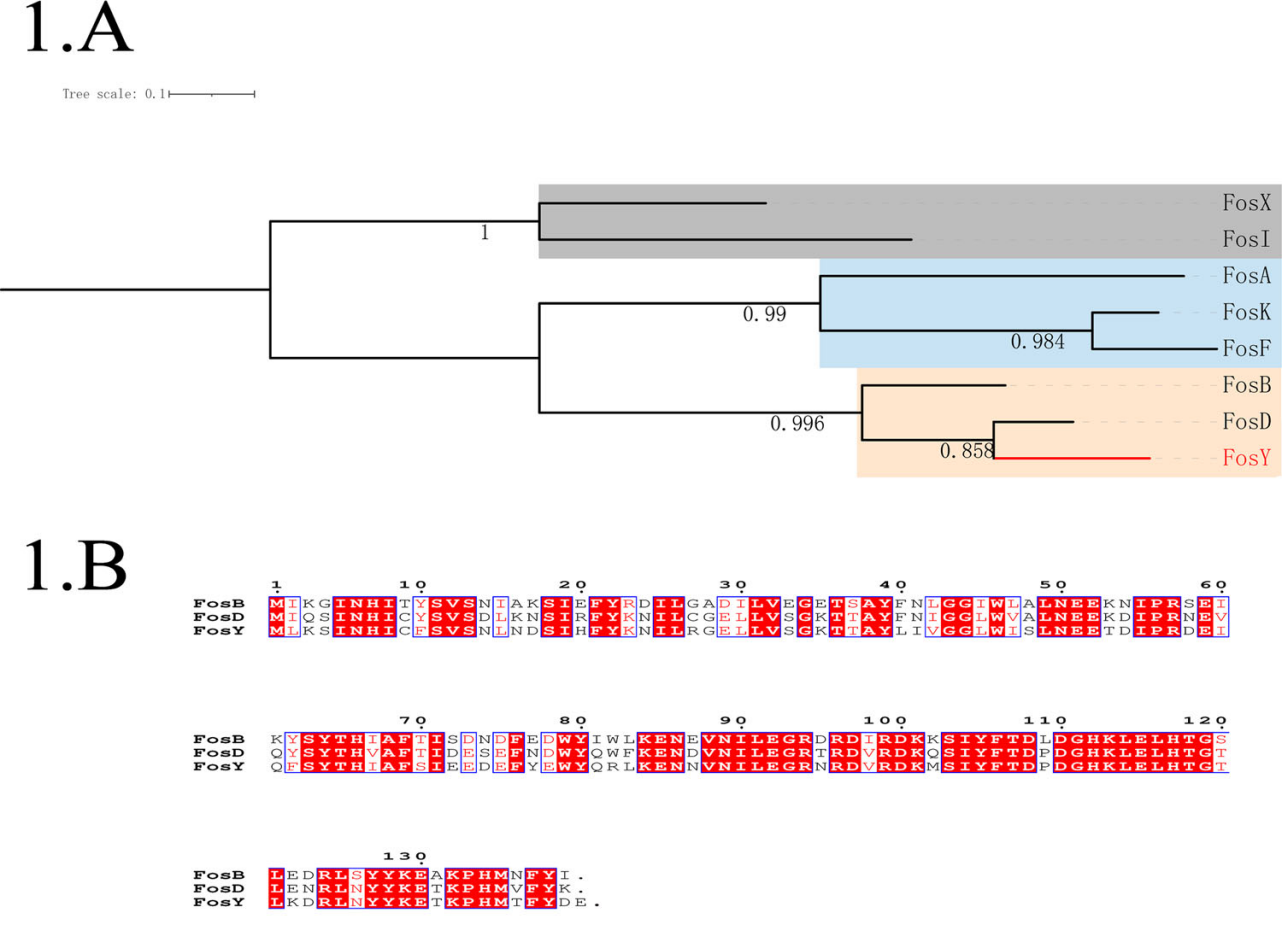
Phylogeny of known fosfomycin-modifying enzymes and amino acid sequence alignment of fosfomycin-modifying enzymes in *Staphylococcus aureus*. (A) Phylogenetic tree obtained for all the identified fos enzymes. Protein sequences were aligned using ClustalW and the tree was generated using MEGA X. The tree is drawn to scale, with branch lengths measured in the number of substitutions per site. The clade containing fosY is highlighted. (B) Amino acid sequence alignment of fosfomycin-modifying enzymes in *Staphylococcus aureus*. Sequence alignment was generated by ClustalW and ESPript 3.0. The same amino acid in the three enzymes is highlighted in red.

To further clarify the genetic relationship of the novel protein FosY, a phylogenetic tree was constructed using the multiple amino acid sequences of other fosfomycin-modifying enzymes (Figure 1(A)). The phylogenetic tree showed that these fosfomycin-modifying enzymes could be differentiated into three groups. FosY and two other fosfomycin-modifying enzymes, that were reported in gram-positive bacteria, were in the same clade. The amino acid sequence of FosY shares 65.9% and 77.5% identity with that of FosB [6] and FosD [7], respectively. A detailed comparison of the amino acid sequences of FosB, FosD, and FosY is shown in Figure 1(B).

### Function of fosY

Fosfomycin susceptibility testing showed that the MIC of the ST1 strain, N12HSA28, was 16 mg/L. Although this strain remained susceptible to fosfomycin, the MIC was 4–32-fold higher than that of other *fosY*-negative ST1 isolates (Table 1). To further confirm the functionality of *fosY* and its contribution to fosfomycin resistance, *fosY* was cloned into pTXΔ and expressed in *S. aureus* RN4220.

The MIC of fosfomycin for the RN4220 pTX-*fosY* transformant was 16 mg/L, whereas the MIC for the pTX16 transformant was 1 mg/L. The MICs of other antibiotics were essentially invariant (Table 2). Therefore, the RN4220 pTX-*fosY* transformant exhibited a 16-fold increase in the MIC of fosfomycin, compared with the RN4220 carrying the empty vector, indicating that *fosY* can decrease fosfomycin susceptibility in *S. aureus*.

**Table 2.** MICs for N28HSA12, cloning strains and recipients.

Strains	MIC (mg/L)					
	FOS	ERY	LEV	GEN	VAN	LZD
N28HSA12	16	>256	0.25	0.5	1	4
RN4220	0.5	0.5	0.25	0.5	1	2
Transformant RN4220 + pTX16	1	0.5	0.25	1	1	2
Transformant RN4220 + pTXΔ-*fosY*	16	0.5	0.25	1	1	2

Abbreviations: ERY: erythromycin; FOS: Fosfomycin; GEN: gentamicin; LEV: levofloxacin; LZD: linezolid; VAN: vancomycin.

### Genetic structure surrounding fosY

**Figure 2.**
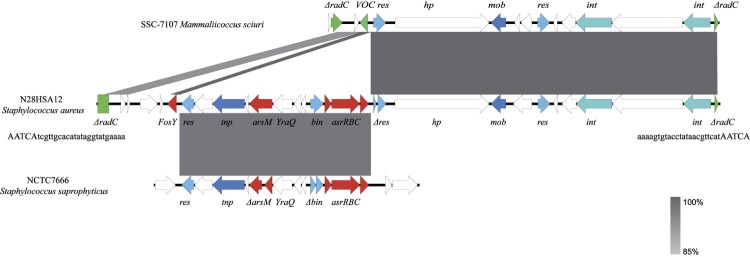
Schematic presentation of *fosY*-carrying genomic island (RI*_fosY_*) in comparison with other sequences. Regions of 100% nucleotide sequence identity are marked in dark grey, while dark grey represents region of 85% nucleotide sequence identity. Arrows indicate the positions and orientations of the genes. The genetic environments of *fosY* in other strains were identical to RI*_fosY_* (99.98% nucleotide identity).

The whole genome of N12HSA28 was sequenced to characterise the genetic environment surrounding *fosY*. The ST1 MRSA strain N12HSA28 contained a 2,833,771-bp circular chromosome in which *fosY* was located. Further analysis showed that *fosY* is located on a 27.6-Kb genomic island inserted into the chromosomal *radC* gene with a 24-bp invert repeat (TCGTTGCACATATAGGTATGAAAA) and a 5-bp direct repeat (AATCA) at both termini (Figure 2). Consequently, we designated this element a “resistance island carrying *fosY*” (RI*_fosY_*). Further genome mining revealed that *fosY*, *arsM*, and a functional operon containing three genes, *asrR*, *asrB*, and *asrC*, colocalised in RI*_fosY_*. The genomic structures of the other three *fosY*-positive strains were compared with the reference RI*_fos_* using Mauve, showing that the genetic environments surrounding *fosY* in all these strains in our study were identical to RI*_fosY_*_._

A comparative genomic analysis was warranted to further our understanding of the origin of RI*_fosY_*. The 15.7-Kb flanking sequences carrying recombinase, integrase, and functionally unknown genes had the highest similarity to the genomic island of *Mammaliicoccus sciuri* strain SSC-7107 collected from a human sample in Pakistan (accession number CP071138), which was inserted into the homologous *radC* gene. The other 9-Kb arsenic-resistance fragment, flanked by recombinases, was similar to the chromosome of *Staphylococcus saprophyticus* strain NCTC7666 isolated from milk ice cream (accession number LR134089). RI*_fosY_* is presumably generated via multiple interspecies recombination events. In addition, *fosY* was homologous to the vicinal oxygen chelate (VOC) family gene in *Mammaliicoccus sciuri* strain SSC-7107 (coverage 77%, identity 100%).

### Global phylogenetic analysis of fosY-positive CC1 *S. aureus*

**Figure 3.**
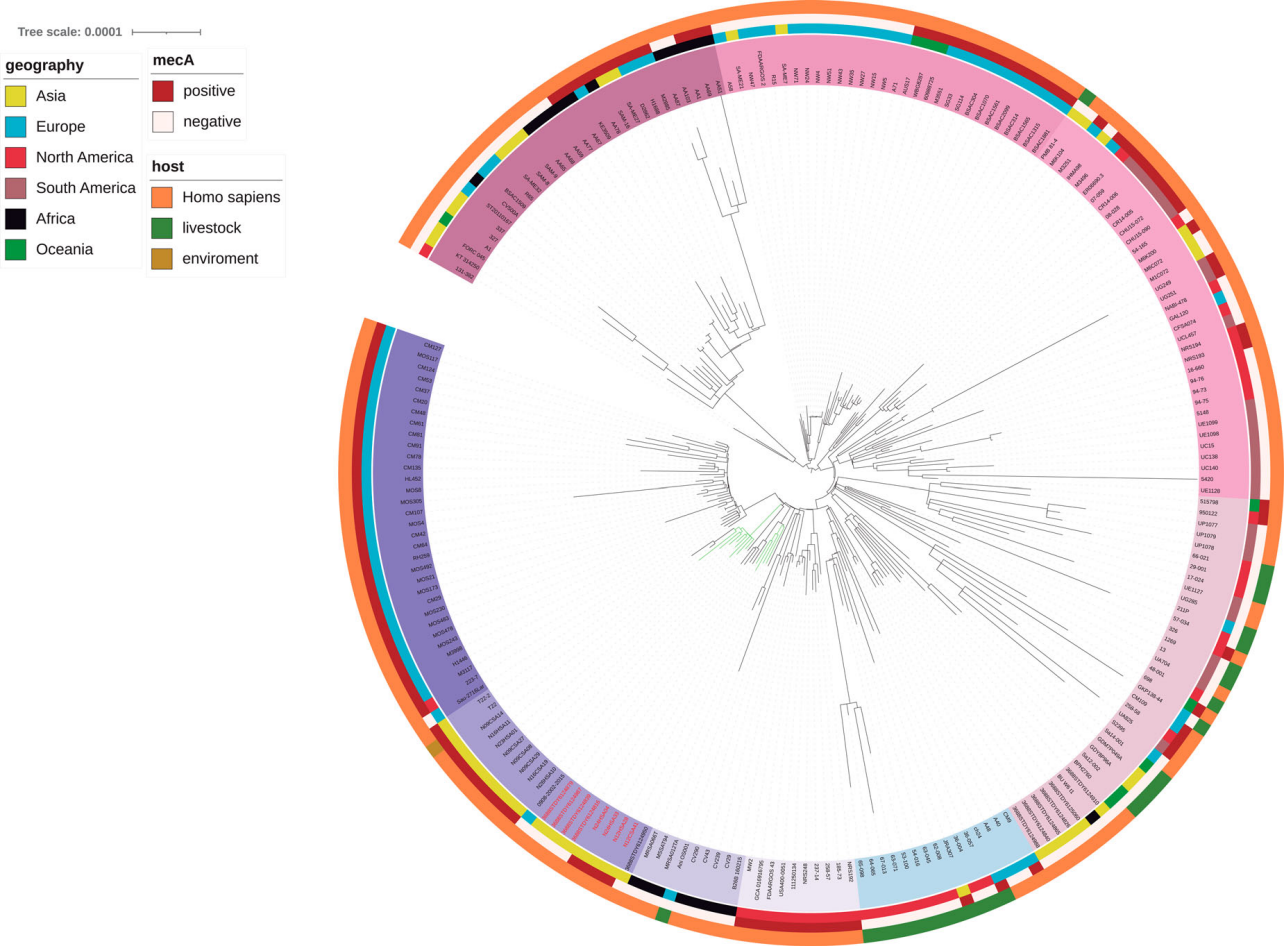
A core-genome global phylogenetic tree of ST1 *Staphylococcus aureus.* The tree is rooted at the midpoint. The labels of *fosY-*positive strains are marked in red. The branches of the strains isolated in this study are marked in green. Major clades are identified based on the coloured backgrounds of the branches. The inner coloured ring indicates the collection region of all the genomes. The next ring indicates *mecA* and the hosts of the isolates are shown in the outermost ring.

To explore the characteristics of the *fosY*-positive ST1 clone in China, a phylogenetic tree was constructed that included 12 CC1 strains (11 ST1 and 1 ST5537), identified in this study, and 206 ST1 strains identified worldwide. Global CC1 clone were classified into some clades, which presented different features. The regions and hosts of the CC1 strains were diverse and were included in the outer circle of the tree. In addition, *mecA* has been identified and labelled in Figure 3.

The global phylogenetic tree showed strains isolated in this study clustered into two subgroups (Figure 3). One of the subgroups included four *fosY* positive MSSA isolates from Thailand. The intact RI*_fosY_* structure carring *fosY* were found in the genome of these four Thailand strains with 99.98% nucleotide identity. All *fosY*-positive strains were adjacent to each other in the phylogenetic tree. In addition, all strains collected in this study are included in the Southeast Asia clade, which is close to the European and African clades.

## Discussion

Recently, fosfomycin has gained attention for the treatment of MRSA infection. Multicentre clinical and experimental studies have demonstrated the clinical efficacy and safety of fosfomycin, in combination with other antibiotics such as imipenem and daptomycin [2,20,21]. Previous studies have focused on the mechanism of fosfomycin resistance in *S. aureus* and have suggested that mutations in chromosomes might be the primary underlying mechanism, as genes that encode the fosfomycin-modifying enzymes are rare in fosfomycin-resistant isolates [22,23]. The discovery of the novel fosfomycin-resistance-related gene, *fosY*, in this study, indicates the importance of fosfomycin-modifying enzyme genes in fosfomycin resistance in *S. aureus*.

Since the identification of fosfomycin-resistance genes in the plasmid of *S. epidermidis* almost 30 years ago [6], several fosfomycin-resistance genes in staphylococci have been reported. Wang et al. reported some *fosB* genes, designated *fosB3* to *fosB6*, in the plasmids of *S. aureus* and *Enterococcus faecium*, which differed from the first *fosB* gene by only 1% to 2% [24,25]. In 2008, the new *fosD* gene was first reported in pTZ2162 from *S. aureus* in Japan [7]. These genes encode modifying enzymes that inactivate fosfomycin [3]. In *Bacillus* species, the homologous FosB enzyme, which belongs to the VOC superfamily [26], catalyses the reaction between bacillithiol and fosfomycin [5], As bacillithiol is also described as a major thiol in staphylococci [27], these acquired fosfomycin-modifying enzymes in *S. aureus* are further supposed as bacillithiol transferase. In this study, we confirmed the sequence of another fosfomycin-modifying enzyme gene, *fosY,* in *S. aureus.* We further showed that *fosY* diverges from the previously described *fosB* and *fosD* genes; meanwhile, the function of *fosY* gene that decreases fosfomycin susceptibility was confirmed in this study. Using phylogenetic analysis, we also showed that FosY clusters with FosB and FosD in the phylogenetic tree, indicating that they share a similar ancestry and we, consequently, identified FosY as a bacillithiol transferase.

Mobile genetic elements can disseminate resistance genes and promote intracellular DNA mobility [28]. A genomic island is a distinct region of a bacterial chromosome that has been acquired via horizontal transfer [28]. It has been reported that transposons, such as Tn554 and Tn559, integrate in the chromosomal *radC* gene in *S. aureus* [29]. In this study, we describe the insertion of a novel 27.6-kb genomic island, RI*_fosY_*_,_ carrying the *fosY* gene. The structure of the island and the *fosY* gene were found to be similar to that in other species, such as *M. sciuri* and *S. saprophyticus* associated with livestock [30–32], implying that RI*_fosY_* may originate and recombine in the microbiome in livestock and then integrate into CC1 *S. aureus*. Therefore, RI*_fosY_* facilitates cross-species gene transfer, and the transfer of RI*_fosY_* to other clones may also be possible. Moreover, a resolvase located upstream of *fosY* may confer the capacity for recombination of this gene. In addition to fosfomycin resistance, the S-adenosylmethionine methyltransferase ArsM and the As(III) efflux system ArsB, which is employed by several bacteria for arsenic tolerance [33,34], are also found in RI*_fosY_*. Arsenic has been found in manure, soil, and effluents of livestock farms [35]. Therefore, the acquisition of RI*_fosY_* may enhance the competitiveness of *S. aureus* to survive in livestock.

*FosY* was identified in the USA400/ST1/SCCmec IV lineage, the most prominent community-associated clone in the USA 20 years ago [8]. To date, ST1 remains one of the most important *S. aureus* lineages in the clinical setting because of its high virulence. Meanwhile, the ST1 clone has been reported to occur sporadically worldwide and has emerged in the clinical setting and livestock in Asia [9,36]. In the global CC1 phylogenetic tree, different clades exhibit different features. Some MRSA strains are clustered in the same clades with some MRSA and MSSA strains distributed crossly. Isolates in some clades show regional aggregation, whereas those in other clades disseminate between continents. Most MSSA strains are located in the intercontinental clades. This indicates that the important lineage ST1 may have various evolutions.

The ST1 strains in our study belong to the same clade, which were mostly collected in Southeast Asia. The phylogenetic tree indicates that this clade has the most recent common ancestor with the European MRSA clade and African MSSA clade. Eight *fosY-*positive strains with similar genetic backgrounds were obtained from Sichuan and Hainan Provinces in Southern China and Thailand, implying that *fosY-*positive strains may be transmitted in these regions. The structures of the *fosY* resistance island in eight strains were basically identical, further proving the dissemination of this *fosY*-positive MRSA clone in Southeast Asia. The *fosY*-positive strains in Thailand were all MSSA, indicating that the acquisition of *fosY*-carrying genomic islands may occur earlier than the *mecA* gene. It is worth noting that the novel *fosY* gene may increase the resistance to fosfomycin in both MSSA and MRSA and disseminate between regions.

Furthermore, some studies have reported ST1 in commercial pig farms, abattoirs, and in companion animals globally [37,38]. In addition, several isolates collected from livestock were included in the evolutionary tree. These livestock-associated strains were almost all MSSA and distributed worldwide and were interspersed with human strains in the same clades, although some studies have reported that the livestock-associated ST1 strains emerged in China [10,39,40]. However, the lack of complete genome information is a limitation to further analyse the relationship between the livestock-associated ST1 in China and the clinical ST1 in our study. All *fosY-*positive strains in our study contained RI*_fosY_*, which is associated with a higher fosfomycin and arsenic resistance. The possibility of these strains infecting livestock must be monitored.

In conclusion, we identified a novel fosfomycin-modifying enzyme-encoding gene, *fosY* located in RI*_fosY_* and originating from other species, in CC1 *S. aureus*, which provides a transferable source of acquired resistance among isolates. Our findings show that a new member of the Fos family confers a decreased susceptibility to fosfomycin in *S. aureus*. As fosfomycin is currently considered a treatment of choice for staphylococcal infections, strategies should be implemented to monitor the spread of these resistant factors.

## Supplementary Material

Supplemental Material

Supplemental Material

## Data Availability

The datasets used and/or analysed during the current study are available from the corresponding author on reasonable request.
