# Supplementary material for: The novel fosfomycin resistance gene fosY is present on a genomic island in CC1 methicillin-resistant Staphylococcus aureus
Source: Emerg Microbes Infect. 2022 Apr 20;11(1):1166–73. doi: 10.1080/22221751.2022.2058421 (PMC9037201; doi:10.1080/22221751.2022.2058421)
Supplement: Supplemental Material [file TEMI_A_2058421_SM9899.docx]

**Supplementary Table1. Summary of the modifying Fos enzymes which have been identified**

| **Determinant** | **Accession number** | **Species** | **Nature of the enzyme** | **Reference** |
| --- | --- | --- | --- | --- |
| FosA | M85195.1 | Serratia marcescens | glutathione-S-transferase | 1^1^ |
| FosB | X54227.1 | Staphylococcus epidermidis | bacillithiol transferase | 2^2^ |
| FosD | AB304512 | Staphylococcus aureus | bacillithiol transferase | 3^3^ |
| FosI | CP003505 | Mycobacterium abscessus | epoxide hydrolase | unpublished |
| FosF | AY294333 | Pseudomonas aeruginosa | glutathione-S-transferase | 4,5^4, 5^ |
| FosK | AB917040 | Acinetobacter soli | glutathione-S-transferase | 6^6^ |
| FosX | AL591981 | Listeria monocytogenes | epoxide hydrolase | 7^7^ |

**Reference**

1. Navas J, León J, Arroyo M et al. Nucleotide sequence and intracellular location of the product of the fosfomycin resistance gene from transposon Tn2921. *Antimicrobial agents and chemotherapy* 1990; **34**: 2016-8.

2. Etienne J, Gerbaud G, Fleurette J et al. Characterization of staphylococcal plasmids hybridizing with the fosfomycin resistance gene fosB. *FEMS microbiology letters* 1991; **68**: 119-22.

3. Nakaminami H, Noguchi N, Nishijima S et al. Characterization of the pTZ2162 encoding multidrug efflux gene qacB from Staphylococcus aureus. *Plasmid* 2008; **60**: 108-17.

4. Yatsuyanagi J, Saito S, Harata S et al. Class 1 integron containing metallo-beta-lactamase gene blaVIM-2 in Pseudomonas aeruginosa clinical strains isolated in Japan. *Antimicrobial agents and chemotherapy* 2004; **48**: 626-8.

5. Yatsuyanagi J, Saito S, Konno T et al. The ORF1 gene located on the class-1-integron-associated gene cassette actually represents a novel fosfomycin resistance determinant. *Antimicrobial agents and chemotherapy* 2005; **49**: 2573.

6. Kitanaka H, Wachino J, Jin W et al. Novel integron-mediated fosfomycin resistance gene fosK. *Antimicrobial agents and chemotherapy* 2014; **58**: 4978-9.

7. Fillgrove KL, Pakhomova S, Newcomer ME et al. Mechanistic diversity of fosfomycin resistance in pathogenic microorganisms. *Journal of the American Chemical Society* 2003; **125**: 15730-1.
